# Unprecedented Pseudotumor Formation in *Granulicatella adiacens* Periprosthetic Joint Infection

**DOI:** 10.1016/j.artd.2026.101961

**Published:** 2026-02-24

**Authors:** Julie Boever, Boris Michael Holzapfel, Wolfgang Böcker, Veronika Kanitz, Maximilian Lerchenberger, Jan Wulf

**Affiliations:** a Department of Orthopaedics and Trauma Surgery, Musculoskeletal University Center Munich (MUM), University Hospital, LMU Munich, Munich, Germany; b Institute of Pathology, Faculty of Medicine, LMU Munich, Munich, Germany

**Keywords:** Periprosthetic joint infection, *Granulicatella adiacens*, Pseudotumor, Hip prosthesis, Immunosuppressive environment

## Abstract

Periprosthetic joint infections remain among the most devastating complications after arthroplasty. While typically caused by common pathogens, rare organisms such as *Granulicatella adiacens* pose unique diagnostic and therapeutic challenges. We describe the first documented case of hip periprosthetic joint infection presenting as giant multilocular pseudotumor in a 68-year-old woman, 14 years after total hip arthroplasty. Despite normal inflammatory markers, aspiration and intraoperative samples identified *G adiacens* by matrix-assisted laser desorption ionization-time of flight. Multimodal surgical treatment with pseudotumor resection, neurolysis, staged component exchange, and targeted antibiotics led to full recovery and infection-free follow-up. This case highlights the need for heightened awareness of atypical pathogens, underscores the value of advanced diagnostics and interdisciplinary management, and raises the hypothesis that pseudotumor environments may facilitate infection.

## Introduction

Periprosthetic joint infections (PJIs) represent a serious complication following joint replacement, often leading to high morbidity and mortality rates. There are a variety of pathogens that can lead to PJIs, most commonly *Staphylococcus aureus* and *Streptococcus spp*. [1] Less common pathogens, as for example *Granulicatella adiacens*, showing fastidious growth requirements, can present unique challenges in both diagnosis and treatment. [2]

*G adiacens* is part of the human oral, urogenital, and gastrointestinal flora and is rarely reported as a human pathogen. [3,4] In this case report, we present a rare instance of a hip prosthesis infection caused by *G adiacens*, which presented with a pseudotumor characterized by a large multilocular, septate, cystic formation on magnetic resonance imaging (MRI). To our knowledge, this is the first report of a pseudotumor associated with *G adiacens* infection in the literature. Furthermore, we conducted a literature review of all 9 known cases of PJI involving *G adiacens* worldwide.

## Case history

In January 2024, a 68-year-old female patient presented to our musculoskeletal university center with progressively increasing swelling of her right thigh. In 2010, she underwent a cementless, ceramic-on-polyethylene right total hip replacement via the lateral approach (Zimmer, Allofit 52 mm shell, Durasul Alpha 32 mm insert, Spotorno 9.0 135° stem, 12/14 taper, Sulox 32mm/-3.5 size S ceramic head) due to hip osteoarthritis in the context of a preexisting hip dysplasia. The postoperative course was complicated by restricted range of motion of the hip, which subsequently led to symptomatic blockades of the vertebral column. At presentation 14 years later, she denied fever or weight loss, but reported night sweating during the past 3 months. Her skin, soft tissues, and the old scar were without clinical signs of inflammation. Laboratory investigations showed a normal value for C-reactive protein (CRP) (0.4 mg/dl; normal range ≤0.5 mg/dl) and a normal leukocyte count (4.68 G/l; normal range 4.0 - 10.4 G/l).

Her medical history included multiple injuries, notably a right anterior cruciate ligament tear and a lateral ligament rupture on the right upper ankle joint, polyarthritis in the fingers and hands, tenovaginitis stenosans, sigmoid diverticulosis, monoclonal gammopathy of undetermined significance, iron deficiency anemia, and a nickel allergy. As long-term medication, the patient takes progesterone (Utrogest), biotin (Priorin), omega-3 fatty acids, and probiotic supplements with active gut bacteria orally, as well as transdermal estradiol (Estreva).

**Figure 1:**
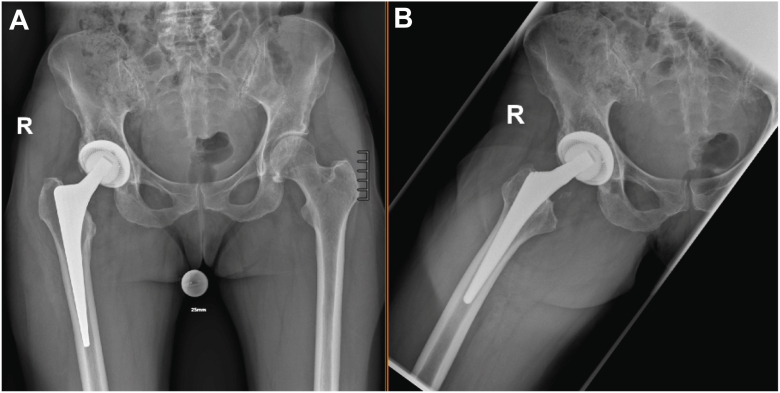
X-rays at the initial presentation in January 2024 showing no evidence of loosening or fracture of the right hip prosthesis. (a) Anterior-posterior pelvic view. (b) Axial view of the right hip according to Lauenstein.

**Figure 2:**
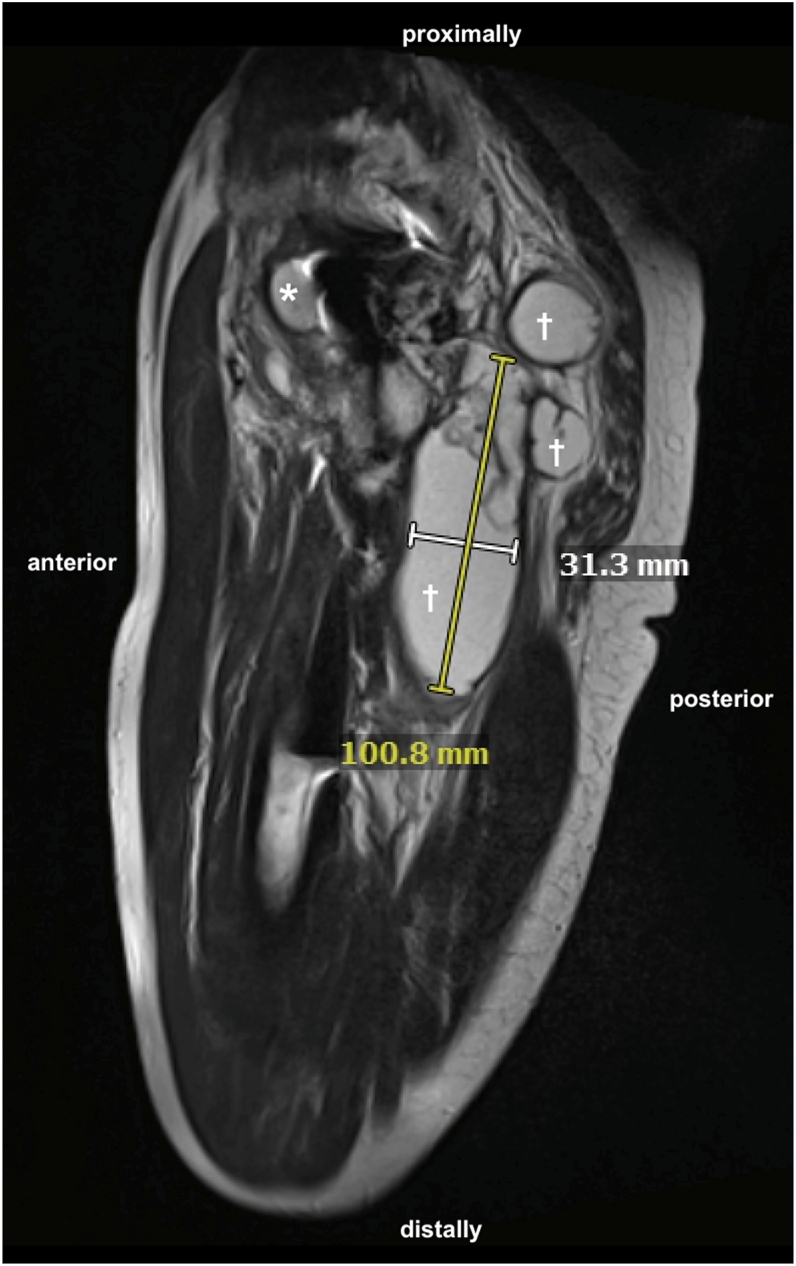
Sagittal view of an MRI with T2 weighting and TSE sequencing of the right proximal thigh in December 2023, revealing a hyperintense multicystic, partly communicating mass measuring up to 100 × 31 mm, located posteriorly (cross) and anteriorly (asterisk) to the hip prosthesis. TSE, turbo spin echo.

**Figure 3:**
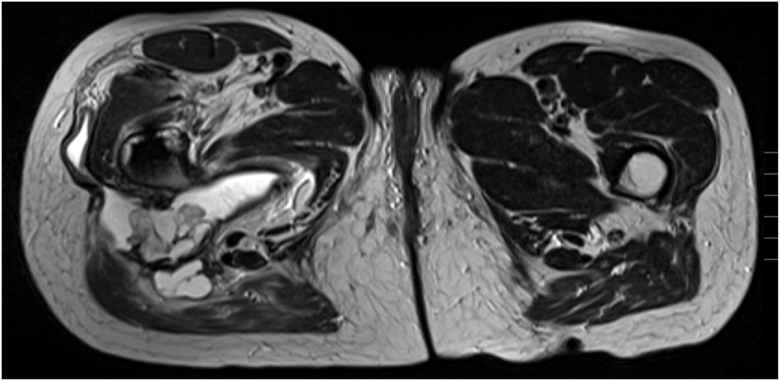
Axial view of an MRI with T2 weighting and TSE sequencing in December 2023 at the level of the pseudocyst in the right thigh, in direct contact with the sciatic nerve. TSE, turbo spin echo.

Radiographic studies did not reveal any signs of prosthesis loosening or periprosthetic fracture (see Fig. 1). However, MRI examinations revealed a large multilocular, septate, cystic formation, communicating with the joint, in direct contact with the sciatic nerve (see Figs. 2 and 3). The blood test for metal ions showed no elevated levels in the serum. Approximately 10 ml of yellowish fluid were aspirated, containing 23,740 leukocytes/μl, with 64% polymorphonuclear cells. The aspiration was tested slightly positive for *G adiacens* using matrix-assisted laser desorption ionization-time of flight (MALDI-TOF) mass spectrometry identification.

Due to the described symptoms and diagnostic findings, surgical intervention was initiated. Before the surgery, an echocardiogram was performed to rule out an endocarditis, and the patient underwent a dental examination during which no signs of acute infections, caries, or inflammation in the oral and maxillofacial region were observed. However, the patient underwent multiple oral treatments in the past, with the latest being a crown replacement of the molars in 2023. Moreover, the patient experienced morning cough with yellow sputum 2-5 times a month for the past 4-5 years, accompanied by repeatedly elevated CRP levels, which have been related to monoclonal gammopathy of undetermined significance by prior physicians. The patient has not taken any antibiotics in the past year.

In March 2024, the patient was admitted to the hospital for surgical treatment. When she arrived, laboratory examinations revealed a slightly elevated value for CRP (1.5 mg/dl) and a normal leukocyte count (5.45 G/l).

**Figure 4:**
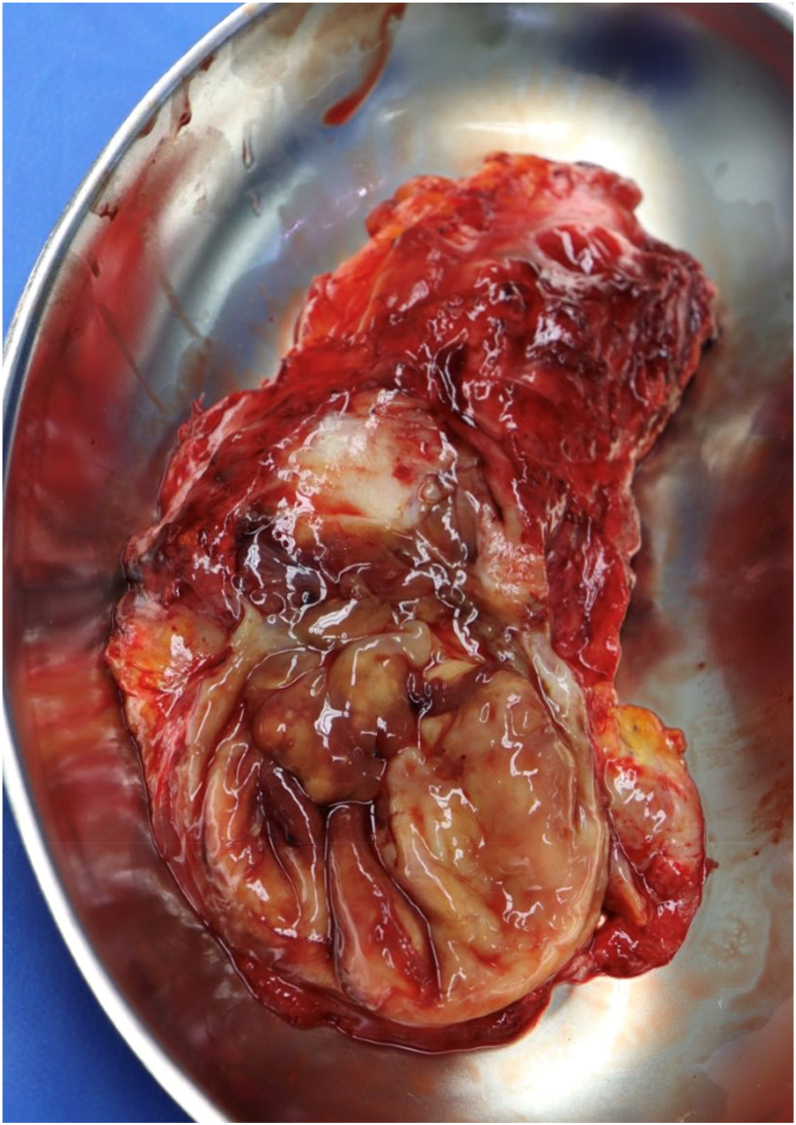
Macroscopic appearance of intraoperatively resected pseudotumor.

**Figure 5:**
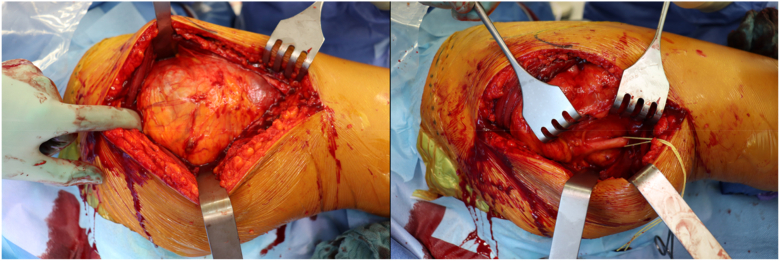
Intraoperative preparation of the tumor (left) via the posterior approach with decompression and neurolysis of the sciatic nerve (right).

**Figure 6:**
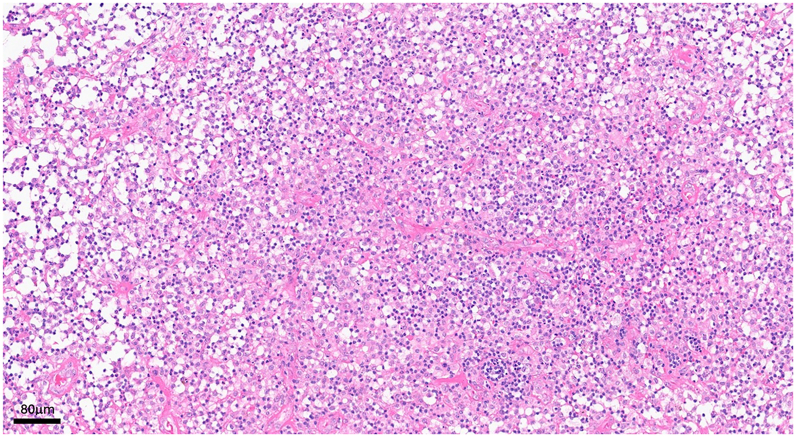
Histopathological analysis of inflamed resected periprosthetic tissue (hematoxylin and eosin staining): Florid granulocytic and fibrinous-exudative as well as chronic-granulating and fibrosing inflammation.

On the day of admission, we resected the pseudotumor and performed a neurolysis of the sciatic nerve, a radical debridement, and a replacement of the inlay as well as the prosthetic head via a posterior approach (see Figs. 4 and 5). This therapeutic approach was chosen because, at that time, it was not possible to definitively determine whether it was an abscess formation or a pseudotumor due to metal debris or trunnionosis, despite normal serum metal ion levels. Furthermore, the microbiologic tests were not entirely conclusive, and the mildly positive test for *G adiacens* could have been attributed to contamination during aspiration. Only after revision surgery, intraoperative samples of the acetabulum were conclusive for infection and showed a persistent infection with *G adiacens*. Therapy with intravenous cefuroxime, 1.5 g, twice daily, was initiated. Following consultation with our antibiotic stewardship team, the treatment was altered to intravenous ampicillin/sulbactam, 3 g, three times daily, on the subsequent day. Since the identified *G adiacens* showed a sensitivity to penicillin G (minimum inhibitory concentration: 0.03 mg/l), the antibiotic therapy could be deescalated to intravenous penicillin G, 5 million IE, four times daily, after 1 week. Histopathological examination of the removed synovia and periprosthetic membranes revealed periprosthetic membranes of the infectious type, classified as type II according to the Krenn-Morawietz classification listed in Table 1 (see Fig. 6). [5]

**Table 1:** Krenn-Morawietz classification to categorize periprosthetic membranes into different types based on histopathological characteristics.

Type	Characteristics
Type I	Neo-synovium/periprosthetic membrane of the abrasion-induced type
Type II	Neo-synovium/periprosthetic membrane of the infectious type
Type III	Neo-synovium/periprosthetic membrane of the combined type
Type IV	Neo-synovium/periprosthetic membrane of the fibrous type

**Figure 7:**
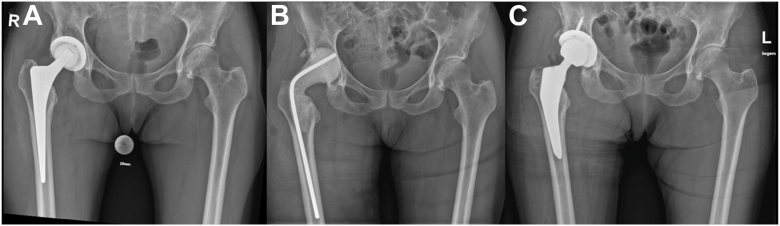
Preoperative (a), spacer (b) and postoperatively (c) pelvic views. Postoperative imaging 2 months after reimplantation reveals no evidence of loosening of the right hip prosthesis.

A week later, a second surgery was performed for radical debridement, removal of the hip arthroplasty, and insertion of an antibiotic-loaded hip joint spacer following resistance testing. Implant removal was technically demanding due to extensive scarring and capsular contracture. The femoral stem was well-fixed and carefully extracted using Moreland chisels and a stem extractor, with meticulous protection of surrounding soft tissues. The acetabular component was also well-integrated and released circumferentially using a Cup-Explant system. Following complete removal, the femoral canal was thoroughly debrided and reamed with flexible reamers. An articulating, antibiotic-loaded cement spacer was then fashioned intraoperatively using PALACOS MV + G bone cement (Heraeus) mixed with vancomycin, with the spacer design adapted to the estimated offset and leg length of the original prosthesis. No more pathogens were detected from samples taken during this operation. The spacer was removed after 15 days with implantation of a new cementless ceramic-on-polyethylene total hip arthroplasty (Aesculap, Plasmafit 56 mm Plus 7 shell stabilized with three anchoring screws, Vitilene 36 mm insert, CoreHip size 4 standard 132° stem, 12/14 taper, Biolox Delta 36 mm size L ceramic head) (see Fig. 7). The stem achieved excellent primary stability through a press-fit fixation, obviating the need for distal anchorage or cement augmentation. This approach was chosen to preserve diaphyseal bone stock, facilitate future revision if necessary, and align with the biological principles of bone integration under stable mechanical conditions. The acetabular reconstruction similarly achieved firm press-fit stability with the addition of three 6.5 mm cancellous screws for supplemental fixation.

The decision to proceed with second-stage reimplantation after a shortened spacer interval of 15 days was made following thorough interdisciplinary evaluation and detailed discussion with the patient. Although a conventional 6-week interval had initially been advised, the patient explicitly requested earlier reimplantation due to limited mobility, and considerable psychological strain. At the time of reassessment, the clinical and microbiological conditions were highly favorable. The patient had received continuous intravenous antibiotic therapy with penicillin G, to which *G adiacens* demonstrated high susceptibility. The soft tissue situation had markedly improved, with no local signs of infection and no additional pathogens had been detected in intraoperative cultures from the first-stage procedure. After review by the institutional antibiotic stewardship team, early reimplantation was deemed clinically acceptable within the context of ongoing targeted antibiotic coverage. The patient provided written informed consent after extensive counseling regarding the risks and benefits of early reimplantation, including the potential for reinfection and mechanical complications.

Intraoperatively, both the acetabular and femoral bone stock were well preserved, and the soft tissue balance was satisfactory.

During her stay, the patient developed no further complications besides herpes infection of the lip, treated with acyclovir ointment. Following the second-stage reimplantation, intravenous penicillin G (5 million IU 4 times daily) was continued until hospital discharge 7 days after surgery. After consultation with microbiology, therapy was transitioned to oral moxifloxacin 400 mg once daily for 12 weeks, chosen for its superior bioavailability compared to oral amoxicillin. The patient was discharged in good general condition for outpatient care, with a follow-up appointment scheduled 2 months after surgery.

**Figure 8:**
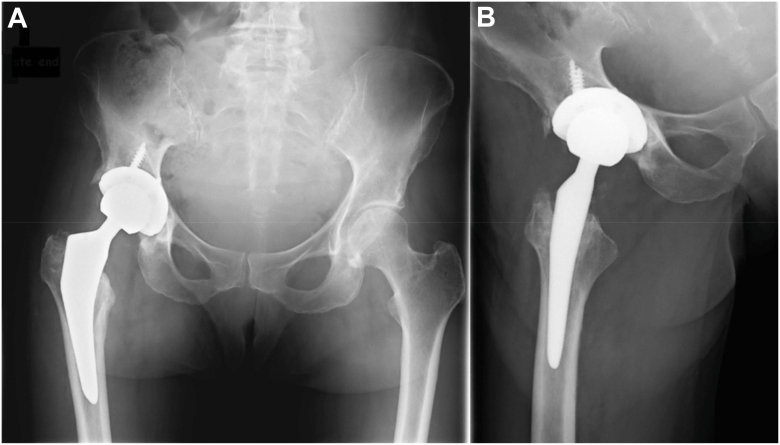
X-rays at the follow-up appointment in June 2024 showing no evidence of loosening or fracture of the right hip prosthesis. (a) Anterior-posterior pelvis view. (b) Axial view of the right hip according to Lauenstein.

At the follow-up appointment in June 2024, there was no sign of persistent bacterial infection of the hip joint. The scar was well healed, and no elevated inflammatory parameters were detectable. The patient was able to walk without the need for an assistive device during the examination. Radiographic studies did not reveal any complications (see Fig. 8).

**Figure 9:**
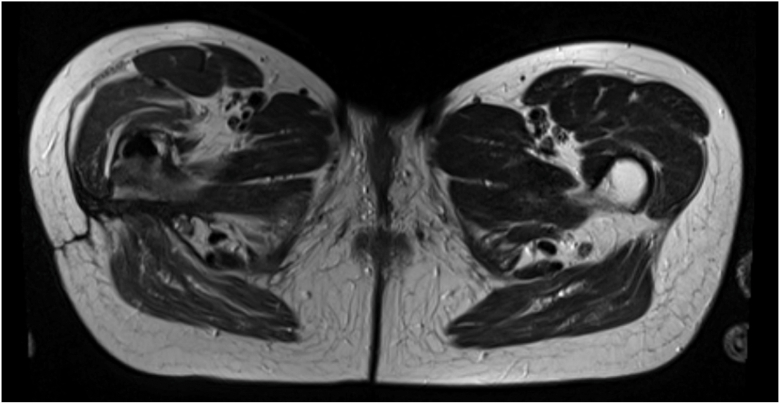
Axial view of an MRI with T2 weighting and TSE sequencing (Metal Artifact Reduction Sequence) at 1-year follow-up at the level of the previous pseudocyst in the right thigh. TSE, turbo spin echo.

At the 1-year follow-up in January 2025, the patient reported being very satisfied without any limitations in daily activities. Laboratory tests showed no evidence of inflammation (leukocytes: 3.4 × 10^3^/μl; hemoglobin: 11.3 g/dl; platelets: 206 × 10^3^/μl; CRP: <0.5 mg/l). An MRI of the pelvis and the right hip revealed no signs of fluid collection or persistent infection (see Fig. 9).

## Discussion

*G adiacens*, formerly *Abiotrophia adiacens*, is a nonmotile, nonsporulating, catalase- and oxidase-negative, facultatively anaerobic Gram-positive coccus. [6] As nutritionally variant streptococci, *G adiacens* is characterized by its fastidious growth needing pyridoxal or cysteine supplementation for growth. [2,6,7]

*G adiacens* is part of the physiological oral, urogenital, and intestinal flora and rarely reported as a human pathogen. [3,4] However, it can cause severe infections in immunocompetent and immunosuppressed patients including endocarditis, osteomyelitis, septic arthritis, keratitis, central nervous system infections, and bacteremia with significant morbidity and mortality. [[8], [9], [10], [11], [12], [13], [14]] In some cases, *G adiacens* infection has been associated with dental procedures, during which the bacterium may enter the bloodstream through transient tissue damage. [[15], [16], [17], [18], [19]] In our case, the patient underwent a dental procedure a few months before the onset of her symptoms, which could potentially be the source of the *G adiacens* infection.

Because of its fastidious growth, an accurate and rapid identification is very difficult. Most often, *G adiacens* is identified by 16S rRNA or MALDI-TOF. [12,20,21] Furthermore, this characteristic can be very challenging for clinical laboratories in antimicrobial susceptibility testing. [22]

To our knowledge, this is the 10th reported case of a *G adiacens–*related PJI worldwide. The other 9 cases are listed in Table 2.

**Table 2:** Overview of the 10 cases of prosthetic joint infection caused by *Granulicatella adiacens* worldwide.

Age (years)	Sex	Prosthetic joint infection	Time between arthroplasty and diagnosis of *G adiacens* (years)	Diagnosis of *G adiacens*	Surgical treatment	Antibiotic treatment (period)	Comorbidities	Dental treatment	Country	Reference
43	Male	Knee arthroplasty	3	16S rRNA	2-stage revision	i.v. amoxicillin + i.v. amikacin (6 weeks), 2 weeks no antibiotic treatment, i.v. amoxicillin + i.v. amikacin + oral rifampicin (2 weeks)	Unknown	Unknown	Switzerland	[23]
55	Male	Knee arthroplasty	10	16S rRNA	2-stage revision	i.v. amoxicillin + i.v. rifampicin (2 weeks), oral amoxicillin + oral rifampicin (3 months)	Diabetes mellitus	Yes	France	[15]
81	Male	Hip arthroplasty	7	MALDI-TOF MS	DAIR, consideration of 2-stage revision	Penicillin G + flucloxacillin (unknown time), i.v. vancomycin + piperacillin/tazobactam + oral fucidic acid (unknown time), i.v. outpatient antimicrobial therapy (OPAT) (1 month), daptomycin + meropenem (6 weeks)	None	Yes	UK	[17]
75	Male	Hip arthroplasty	4	16S rRNA	2-stage revision	Oral amoxicillin + clindamycin (6 months)	High blood pressure, ankylosing spondylitis, sleep apnea	Yes	France	[16]
65	Male	Knee arthroplasty	2	MALDI-TOF MS	1-stage revision	Oral rifampicin + clindamycin (6 months)	Psoriasis, chronic alcoholism, esophagitis	No	France	[16]
44	Female	Hip arthroplasty	10	MALDI-TOF MS + 16S rRNA	DAIR	i.v. imipenem/cystatin + oral ciprofloxacin (1 month), oral amoxicillin + oral ciprofloxacin (5 months)	Unknown	No	France	[16]
64	Male	Knee arthroplasty	7	16S rRNA	2-stage revision	i.v. ertapenem (6 weeks), cefazolin (3 days)	Unknown	Yes	USA	[18]
65	Male	Knee arthroplasty	2	Unknown	DAIR	i.v. vancomycin (unknown time), i.v. clindamycin (6 weeks)	Unknown	Unknown	UK	[24]
79	Male	Hip arthroplasty	3	16S rRNA	2-stage revision	i.v. penicillin G (6 weeks)	Pre-existing cardiac comorbidities with PTCA	Yes	Australia	[19]
68	Female	Hip arthroplasty	14	MALDI-TOF MS	2-stage revision	i.v. cefuroxime (1 day), i.v. ampicillin/sulbactam (5 days), i.v. penicillin G (3 weeks), oral moxifloxacin (2 months)	Multiple injuries, polyarthritis in the fingers and hands, sigmoid diverticulosis, MGUS, iron deficiency anemia	Yes	Germany	

MALDI-TOF MS, matrix-assisted laser desorption ionization-time of flight mass spectrometry; DAIR, Debridement, antibiotics and implant retention; MGUS, monoclonal gammopathy of undetermined significance; i.v., intravenously; PTCA, percutaneous transluminal coronary angioplasty. [[15], [16], [17], [18], [19],^23^,^24^].

Considering these 10 cases of PJI with *G adiacens*, at least 6 patients received a prior dental treatment, which could be causative. German guidelines recommend antibiotic prophylaxis prior to invasive dental treatment for patients with hip or knee replacements with a single dose of oral amoxicillin 2 g as “of label use”, or in case of an allergy clindamycin 600 mg, in accordance with the widely accepted endocarditis prophylaxis. [25,26] The American Academy of Orthopaedic Surgeons does not recommend routine prophylactic antibiotic therapy for patients with hip and knee prostheses before dental procedures. [27] Considering the prolonged treatment with high morbidity and mortality, as well as the high financial costs associated with prosthetic joint infection compared to a single dose of antibiotic therapy, an antibiotic prophylaxis after careful benefit-risk analysis could possibly reduce the number of PJI in specific patients.

As *G adiacens* is difficult to diagnose, we believe that many infections remain unrecognized. It requires close collaboration between various departments, including orthopaedics, microbiology, radiology, and infectious disease specialists.

In patients with prosthetic joint infections, it is essential to consider rare pathogens such as *G adiacens*, particularly when standard tests return negative results.

Furthermore, pseudotumors in joint prosthesis primarily form as a reaction to metallic debridement in metal-on-metal implants, which triggers an immune response to these particles. [[28], [29], [30]] However, our patient had a ceramic-polyethylene articulation, which is associated with a lower incidence of pseudotumors. [31] Trunnionosis remains a possibility due to metal reactions, although metal ions were not elevated in our case. [32] Other reports highlight that pseudotumors can occur with normal blood ion levels, emphasizing the role of periprosthetic metal accumulation. [33,34] Studies show a correlation between high metal ion levels and osteolysis or aseptic loosening, but not specifically with pseudotumors. [35] Additionally, there are documented cases of periprosthetic pseudotumors with positive swabs for fungal infections identifying *Candida parapsilosis*, *Candida albicans*, and for bacterial infections *Ruminococcus gnavus*. [[36], [37], [38]] A plausible hypothesis is that pseudotumors, which can be induced by wear particles from prosthesis, increase the risk of periprosthetic infection by creating an immunosuppressive environment that enhances bacterial growth and persistence. [[39], [40], [41]] Interestingly, MRI of the patient’s right hip in 2017, performed due to a single acute pain exacerbation, did not reveal any pathologies. Therefore, we hypothesize that the formation of the pseudotumor took place after 2017. At this point, further research and a comprehensive review are needed to validate this hypothesis.

## Summary

In conclusion, *G adiacens*, a rare but significant pathogen, poses challenges in both diagnosis and treatment. Accurate identification often necessitates advanced techniques like 16S rRNA sequencing or MALDI-TOF mass spectrometry. Close interdisciplinary collaboration is essential. To ensure rapid and appropriate treatment, clinicians should also consider rare pathogens. In the context of periprosthetic pseudotumors, infections should be evaluated additionally to metallosis and trunnionosis.

## Ethical approval and consent to participate

The study was carried out in accordance with the Declaration of Helsinki. The patient was informed that data concerning the case would be submitted for publication, and she provided consent. Furthermore, we are grateful for the patient's consent and help with gathering all the information presented.

## Conflict of interest

The authors declare there are no conflicts of interest.

For full disclosure statements refer to https://doi.org/10.1016/j.artd.2026.101961.

## Informed patient consent

The author(s) confirm that written informed consent has been obtained from the involved patient(s) or if appropriate from the parent, guardian, power of attorney of the involved patient(s); and, they have given approval for this information to be published in this case report (series).

## Declaration of Generative AI and AI-Assisted Technologies in the Writing Process

We acknowledge the use of Open AI for language refurbishing of the first draft of the manuscript.

## CRediT authorship contribution statement

**Julie Boever:** Writing – original draft, Visualization, Supervision, Project administration, Methodology, Investigation, Formal analysis, Data curation, Conceptualization. **Boris Michael Holzapfel:** Writing – original draft, Supervision, Project administration, Methodology, Investigation, Formal analysis, Conceptualization. **Wolfgang Böcker:** Writing – review & editing, Formal analysis, Conceptualization. **Veronika Kanitz:** Writing – review & editing, Visualization, Conceptualization. **Maximilian Lerchenberger:** Writing – review & editing, Investigation, Formal analysis. **Jan Wulf:** Writing – review & editing, Methodology, Investigation, Formal analysis, Data curation, Conceptualization.

## Appendix A Supplementary data

Conflict of Interest Statement for Böcker

Conflict of Interest Statement for Boever

Conflict of Interest Statement for Holzapfel

Conflict of Interest Statement for Kanitz

Conflict of Interest Statement for Lerchenberger

Conflict of Interest Statement for Wulf

## References

[1] Zardi E.M., Franceschi F. (2020). Prosthetic joint infection. A relevant public health issue. J Infect Public Health.

[2] Ruoff K.L. (1991). Nutritionally variant streptococci. Clin Microbiol Rev.

[3] Aas J.A., Paster B.J., Stokes L.N., Olsen I., Dewhirst F.E. (2005). Defining the normal bacterial flora of the oral cavity. J Clin Microbiol.

[4] Villmones H.C., Svanevik M., Ulvestad E., Stenstad T., Anthonisen I.L., Nygaard R.M. (2022). Investigating the human jejunal microbiota. Sci Rep.

[5] Krenn V., Morawietz L., Perino G., Kienapfel H., Ascherl R., Hassenpflug G.J. (2014). Revised histopathological consensus classification of joint implant related pathology. Pathol Res Pract.

[6] Collins M.D., Lawson P.A. (2000). The genus Abiotrophia (Kawamura et al.) is not monophyletic: proposal of Granulicatella gen. nov., Granulicatella adiacens comb. nov., Granulicatella elegans comb. nov. and Granulicatella balaenopterae comb. nov. Int J Syst Evol Microbiol.

[7] Christensen J.J., Facklam R.R. (2001). Granulicatella and Abiotrophia species from human clinical specimens. J Clin Microbiol.

[8] Adam E.L., Siciliano R.F., Gualandro D.M., Calderaro D., Issa V.S., Rossi F. (2015). Case series of infective endocarditis caused by Granulicatella species. Int J Infect Dis.

[9] Hoen B., Alla F., Selton-Suty C., Béguinot I., Bouvet A., Briançon S. (2002). Changing profile of infective endocarditis: results of a 1-year survey in France. Jama.

[10] Heath C.H., Bowen S.F., McCarthy J.S., Dwyer B. (1998). Vertebral osteomyelitis and discitis associated with Abiotrophia adiacens (nutritionally variant streptococcus) infection. Aust N Z J Med.

[11] Hepburn M.J., Fraser S.L., Rennie T.A., Singleton C.M., Delgado B. (2003). Septic arthritis caused by Granulicatella adiacens: diagnosis by inoculation of synovial fluid into blood culture bottles. Rheumatol Int.

[12] Woo P.C., Fung A.M., Lau S.K., Chan B.Y., Chiu S.K., Teng J.L. (2003). Granulicatella adiacens and Abiotrophia defectiva bacteraemia characterized by 16S rRNA gene sequencing. J Med Microbiol.

[13] Al-Lozi A., Cai S., Chen X., Perez V.L., Venkateswaran N. (2022). Granulicatella Adiacens as an unusual cause of microbial Keratitis and endophthalmitis: a case series and literature review. Ocul Immunol Inflamm.

[14] Cerceo E., Christie J.D., Nachamkin I., Lautenbach E. (2004). Central nervous system infections due to Abiotrophia and Granulicatella species: an emerging challenge?. Diagn Microbiol Infect Dis.

[15] Mougari F., Jacquier H., Berçot B., Hannouche D., Nizard R., Cambau E. (2013). Prosthetic knee arthritis due to Granulicatella adiacens after dental treatment. J Med Microbiol.

[16] Quénard F., Seng P., Lagier J.C., Fenollar F., Stein A. (2017). Prosthetic joint infection caused by Granulicatella adiacens: a case series and review of literature. BMC Musculoskelet Disord.

[17] Aweid O., Sundararajan S., Teferi A. (2016). Granulicatella adiacens prosthetic hip joint infection after dental treatment. JMM Case Rep.

[18] Pingili C., Sterns J., Jose P. (2017). First case of prosthetic knee infection with Granulicatella adiacens in the United States. IDCases.

[19] Badrick T.C., Nusem I., Heney C., Sehu M. (2021). Granulicatella adiacens: an uncommon diagnosis of prosthetic hip joint infection. A case report with review of the literature. IDCases.

[20] Kawamura Y., Hou X.G., Sultana F., Liu S., Yamamoto H., Ezaki T. (1995). Transfer of Streptococcus adjacens and Streptococcus defectivus to Abiotrophia gen. nov. as Abiotrophia adiacens comb. nov. and Abiotrophia defectiva comb. nov., respectively. Int J Syst Bacteriol.

[21] Ratcliffe P., Fang H., Thidholm E., Boräng S., Westling K., Özenci V. (2013). Comparison of MALDI-TOF MS and VITEK 2 system for laboratory diagnosis of Granulicatella and Abiotrophia species causing invasive infections. Diagn Microbiol Infect Dis.

[22] Alberti M.O., Hindler J.A., Humphries R.M. (2015). Antimicrobial susceptibilities of Abiotrophia defectiva, Granulicatella adiacens, and Granulicatella elegans. Antimicrob Agents Chemother.

[23] Riede U., Graber P., Ochsner P.E. (2004). Granulicatella (Abiotrophia) adiacens infection associated with a total knee arthroplasty. Scand J Infect Dis.

[24] Narayana Murthy S., Srinivasan S.H., Archunan M., Cutts S. (2021). Prosthetic knee joint infection by an unusual organism following acupuncture treatment. Acupunct Med.

[25] Heller K.-D., Perka C., Renz N. Handlungsempfehlung für die Antibiotikaprophylaxe bei zahnmedizinischen Eingriffen – ein Update. https://www.ae-germany.com/ae-blog/antibiotikaprophylaxe-bei-zahnmedizinischen-eingriffen-ein-update.

[26] Wilson W., Taubert K.A., Gewitz M., Lockhart P.B., Baddour L.M., Levison M. (2007). Prevention of infective endocarditis: guidelines from the American heart Association: a guideline from the American heart Association rheumatic fever, endocarditis, and Kawasaki Disease Committee, council on cardiovascular Disease in the young, and the council on clinical cardiology, council on cardiovascular surgery and Anesthesia, and the quality of care and Outcomes Research Interdisciplinary Working Group. Circulation.

[27] Watters W., Rethman M.P., Hanson N.B., Abt E., Anderson P.A., Carroll K.C. (2013). Prevention of orthopaedic implant infection in patients undergoing dental procedures. J Am Acad Orthop Surg.

[28] van der Merwe J.M. (2021). Pseudotumors in total joint arthroplasty. JBJS Rev.

[29] Stahnke J.T., Sharpe K.P. (2015). Pseudotumor Formation in a metal-on-polyethylene total hip arthroplasty due to trunnionosis at the head-neck taper. Surg Technol Int.

[30] Hart A.J., Satchithananda K., Liddle A.D., Sabah S.A., McRobbie D., Henckel J. (2012). Pseudotumors in association with well-functioning metal-on-metal hip prostheses: a case-control study using three-dimensional computed tomography and magnetic resonance imaging. J Bone Joint Surg Am.

[31] Das D.H., van der Weegen W., Wullems J.A., Brakel K., Sijbesma T., Nelissen R.G. (2016). Periprosthetic pathology in 'at risk' ceramic-on-polyethylene total hip arthroplasty: a clinical study using MARS-MRI in 50 patients. Hip Int.

[32] Mistry J.B., Chughtai M., Elmallah R.K., Diedrich A., Le S., Thomas M. (2016). Trunnionosis in total hip arthroplasty: a review. J Orthop Traumatol.

[33] Krishnan H., Magnussen A., Sharma A., Skinner J. (2015). Metal on metal total hip arthroplasty and a large groin mass: not always adverse reaction to metallic debris. Int J Surg Case Rep.

[34] Lohmann C.H., Meyer H., Nuechtern J.V., Singh G., Junk-Jantsch S., Schmotzer H. (2013). Periprosthetic tissue metal content but not serum metal content predicts the type of tissue response in failed small-diameter metal-on-metal total hip arthroplasties. J Bone Joint Surg Am.

[35] Manfreda F., Bufi E., Florio E.F., Ceccarini P., Rinonapoli G., Caraffa A. (2021). Osteolysis in total hip arthroplasty in relation to metal ion release: comparison between monolithic prostheses and different modularities. World J Orthop.

[36] Marcomini M.P., Iqbal J., Bennett D. (2023). Rare pseudotumor in ceramic-on-ceramic total hip replacement with concomitant fungal periprosthetic joint infection: a case report. Am J Case Rep.

[37] Artiaco S., Ferrero A., Boggio F., Colzani G. (2013). Pseudotumor of the hip due to fungal prosthetic joint infection. Case Rep Orthop.

[38] Fernández-Caso B., Domingo García D., Domingo L.C., Ampuero J.C. (2017). Ruminococcus gnavus infection of a metal-on-metal hip arthroplasty resembling a pseudo-tumour in a 72 year-old woman with no intestinal symptoms. Enferm Infecc Microbiol Clin.

[39] Leal J., Holland C.T., Cochrane N.H., Seyler T.M., Jiranek W.A., Wellman S.S. (2024). The relationship between pseudotumours and infected complications in patients who have undergone metal-on-metal total hip arthroplasty. Bone Joint J.

[40] Anwar H.A., Aldam C.H., Visuvanathan S., Hart A.J. (2007). The effect of metal ions in solution on bacterial growth compared with wear particles from hip replacements. J Bone Joint Surg Br.

[41] Hosman A.H., van der Mei H.C., Bulstra S.K., Busscher H.J., Neut D. (2010). Effects of metal-on-metal wear on the host immune system and infection in hip arthroplasty. Acta Orthop.

